# Low levels of *IGFBP7* expression in high-grade serous ovarian carcinoma is associated with patient outcome

**DOI:** 10.1186/s12885-015-1138-8

**Published:** 2015-03-17

**Authors:** Karen Gambaro, Michael CJ Quinn, Katia Y Cáceres-Gorriti, Rebecca S Shapiro, Diane Provencher, Kurosh Rahimi, Anne-Marie Mes-Masson, Patricia N Tonin

**Affiliations:** 1 Department of Human Genetics, McGill University, Montreal, H3A 1B1 Canada; 2 Centre de recherche du Centre hospitalier de l’Université de Montréal/Institut du cancer de Montréal, Montreal, H2X 0B9 Canada; 3 Department of Obstetric-Gynecology, Université de Montréal, Montreal, H2L 4M1 Canada; 4 Department of Pathology, Université de Montréal, Montreal, H3C 3J7 Canada; 5 Department of Medicine, Université de Montréal, Montreal, H3C 3J7 Canada; 6 The Research Institute of the McGill University Health Centre, Montreal, H4A 3J1 Canada; 7 Department of Medicine, McGill University, Montreal, H3G 1A4 Canada; 8 Research Institute of the McGill University Health Centre, 1001 Decarie Boulevard, Site Glen Pavillion Block E, Cancer Research Program E026217 (cubicle E), Montreal, Quebec H4A 3J1 Canada

**Keywords:** IGFBP7, Epithelial ovarian cancer, Gene expression, Migration, Tissue microarray, Patient outcome

## Abstract

**Background:**

Insulin-like growth factor binding protein 7 (*IGFBP7*) has been suggested to act as a tumour suppressor gene in various human cancers, yet its role in epithelial ovarian cancer (EOC) has not yet been investigated. We previously observed that *IGFBP7* was one of several genes found significantly upregulated in an EOC cell line model rendered non-tumourigenic as consequence of genetic manipulation. The aim of the present study was to investigate the role of IGFBP7 in high-grade serous ovarian carcinomas (HGSC), the most common type of EOC.

**Methods:**

We analysed IGFBP7 gene expression in 11 normal ovarian surface epithelial cells (NOSE), 79 high-grade serous ovarian carcinomas (HGSC), and seven EOC cell lines using a custom gene expression array platform. *IGFBP7* mRNA expression profiles were also extracted from publicly available databases. Protein expression was assessed by immunohistochemistry of 175 HGSC and 10 normal fallopian tube samples using tissue microarray and related to disease outcome. We used EOC cells to investigate possible mechanisms of gene inactivation and describe various *in vitro* growth effects of exposing EOC cell lines to human recombinant IGFBP7 protein and conditioned media.

**Results:**

All HGSCs exhibited *IGFBP7* expression levels that were significantly (p = 0.001) lower than the mean of the expression value of NOSE samples and that of a whole ovary sample. *IGFBP7* gene and protein expression were lower in tumourigenic EOC cell lines relative to a non-tumourigenic EOC cell line. None of the EOC cell lines harboured a somatic mutation in *IGFBP7*, although loss of heterozygosity (LOH) of the *IGFBP7* locus and epigenetic methylation silencing of the *IGFBP7* promoter was observed in two of the cell lines exhibiting loss of gene/protein expression. *In vitro* functional assays revealed an alteration of the EOC cell migration capacity. Protein expression analysis of HGSC samples revealed that the large majority of tumour cores (72.6%) showed low or absence of IGFBP7 staining and revealed a significant correlation between IGFBP7 protein expression and a prolonged overall survival (p = 0.044).

**Conclusion:**

The low levels of *IGFPB7* in HGSC relative to normal tissues, and association with survival are consistent with a purported role in tumour suppressor pathways.

**Electronic supplementary material:**

The online version of this article (doi:10.1186/s12885-015-1138-8) contains supplementary material, which is available to authorized users.

## Background

Epithelial ovarian cancer (EOC) is the most lethal gynecological cancer in the developed world [1,2]. The high mortality rate (>70%) has been attributed to the advanced stage at diagnosis of most cases and the high relapse rate to paclitaxel/carboplatin chemotherapy following cytoreductive surgery, which is the standard of care for patients [3,4]. EOC is classified into the major histological subtypes referred to as serous, mucinous, endometrioid, clear cell or undifferentiated, based on the morphology of the tumour cells, and assigned a tumour grade according to degree of differentiation where high-grade serous carcinoma (HGSC) represents the largest proportion (up to 70%) of EOC cases [5,6]. Molecular genetic profiling suggests that HGSC is a disease distinct from the other histotypes [7] with over 95% harbouring somatic *TP53* mutations and extensive genomic anomalies [8-11]. With the exception of high-grade endometrioid carcinomas (HGEC), which appear to overlap in their molecular genetic features with HGSC, the less common histotypes are each distinguishable from HGSC based on somatic mutations occurring in specific genes and gene expression profiles [12-14].

Our group has focused on investigating somatic molecular genetic events associated with tumour suppressor pathways affected in HGSC [15-18]. Towards this goal, we have started characterizing the genes reprogrammed in the context of a tumourigenic OV90 EOC cell line rendered non-tumourigenic as a consequence of a unique complementation assay involving the transfer of normal chromosomal fragments [15,16,18]. OV90, derived from a long-term passage of undifferentiated adenocarcinoma of malignant ovarian ascites, exhibits the molecular genetic characteristics of HGSC, which includes the presence of a somatic *TP53* mutation and complex genomic rearrangements overlapping the spectrum of anomalies observed in HGSCs [15,19]. The non-tumourigenic hybrids displayed an altered cell morphology, a reduced capacity for colonies in soft agarose assays, inability to form spheroids in culture assays, and were unable to form tumours after injection into both subcutaneous and intraperitoenal sites in nude mice [15]. A comparative analysis of transcriptomes from the parental tumourigenic OV90 cell line, with each non-tumourigenic genetically derived hybrid, identified a number of genes exhibiting differential expression, some of which have been shown to be implicated in EOC and other cancers [15,17,20,21]. The non-tumourigenic hydrids each acquired a unique spectrum of chromosome 3 genes as a consequence of the complementation assay [15]. However, they all shared in common a transferred 3q12-pter interval, which contained a number of interesting candidates posted to elicit tumour suppressor pathways, including *VGLL3* [15,16,18]. Insulin-like growth factor binding protein 7 (*IGFBP7*), a gene suspected to play a role in tumour suppressor pathways in various cancer types but not extensively studied in EOC, was among the list of genes which were reprogrammed as a consequence of tumour suppression in our OV90 cell line model [15,22-27].

*IGFBP7* (*IGFBP-related protein-1* or *MAC25*) is localized to chromosome 4q12 and encodes a secreted IGFBP-related protein, a member of the IGFBP family, that binds to IGF-I and IGF-II with low affinity, and binds to insulin and activin with higher affinity [28-30]. In various cancer types, *IGFBP7* has been implicated in cellular processes including cell differentiation, cell adhesion, angiogenesis, cell growth and survival, senescence and apoptosis [23,27,31-34]. The study of *IGFBP7* in a variety of cancers, including breast, thyroid, lung, prostate, colorectal, gastric, pancreatic and liver cancer has suggested a role of a tumour suppressor gene [22,24,26,27,35-38]. In each of these cancer types or cell lines, *IGFBP7* was shown down-regulated and in some cases, loss of heterozygosity (LOH), gene deletion or DNA methylation were postulated as a mechanism of inactivation to affect the gene expression [25-27,37,39-43]. *IGFBP7* expression has been shown to be inversely correlated with tumour grade and stage in hepatocellular carcinoma and lung cancer, and has been associated with favourable outcomes in breast, pancreatic, colorectal and liver cancer patients [26,35-38,40,42,44,45]. The emerging role of *IGFBP7* in the development and prognosis of a variety of cancer types is interesting given our observations that gene expression was up-regulated in our genetically modified non-tumourigenic OV90 cell line hybrids, as this would support a role in the tumour suppressor phenotype in this model.

In this report, we describe the gene and protein expression profile *IGFBP7* in normal ovarian surface epithelial cells and fallopian tube samples respectively, as well as in HGSC samples and relate our findings to disease outcome. We focus on HGSC, as this is the most common subtype of EOC. We report the gene and protein expression profile of various well-characterized EOC cell lines, and investigate possible mechanisms of gene inactivation. We also describe various *in vitro* growth effects of exposing EOC cell lines to human recombinant IGFBP7 protein and conditioned media (CM) derived from IGFBP7 protein expressing cells. To our knowledge this is the first report of the expression profile of IGFBP7 in HGSC. We observed overall low or absent expression of IGFBP7 gene and protein relative to normal tissues, and a significant correlation with decreased protein expression in HGSC samples and prolonged overall survival. Our findings combined with dysregulation in a genetic modified ovarian cancer cell line model rendered non-tumourigenic which resulted in the up-regulation of IGFBP7, supports a role in tumour suppressor pathways.

## Methods

### Ethics statement

Written informed consent was obtained from all subjects providing tumour, and associated clinical information, and normal tissue samples that were collected with informed written consent from participants undergoing surgeries performed at the Centre hospitalier de l’Université de Montréal (CHUM) - Hôpital Notre-Dame as part of the tissue and clinical banking activities of the Banque de tissus et de données of the Réseau de recherche sur le cancer (RRCancer) of the Fonds de Recherche du Québec – Santé (FRQS) as described [46] and in accordance with guidelines and approval established by the institutional ethical review boards of the CHUM ethics committee.

### Tumour and normal tissue specimens

Disease stage was assigned by a gynecologic-oncologist, and tumour grade and histopathological subtypes were assigned by a gynecologic-oncologist pathologist according to the criteria established by the International Federation of Gynecology and Obstetrics. Normal tissues samples represent fallopian tubes or ovaries were also evaluated by a gynecologic-oncologist pathologist.

### EOC cell lines

The EOC cell lines and their culture conditions have been described previously [19,46] Briefly, they were established from the malignant ovarian tumours (TOV) and ascites (OV) from patients who had not undergone radiation treatment or chemotherapy prior to surgery and represent different subtypes: undifferentiated adenocarcinoma (OV90), HGEC (TOV112D), a low-grade serous carcinoma (TOV81D), HGSC (TOV1946, OV1946, and TOV2223), and a clear cell carcinoma (TOV21G), where TOV1946 and OV1946 were derived from malignant ovarian ascites (OV1946) or tumour (TOV1946) from the same patient. The non-tumourigenic chromosome 3 transfer radiation hybrids RH-5, RH-6 and RH-10 were derived by fusing a neomycin clone of OV90 (OV90 neo^r^), and an irradiated B78MC166 mouse cell line containing human chromosome 3 as described previously [15].

### Expression microarray analyses

The *IGFBP7* expression values associated with probe set *201162_at* from OV90 neo^r^ (a neomycin clone of the OV90) and the three non-tumourigenic hybrids (RH5, RH6, RH10) were extracted from Affymetrix U133Plus2 GeneChip derived data that was MAS5 generated and normalized as previously described [15]. The expression value of the same probe set from the primary cultures from NOSE (n = 17), the HGEC (n = 7) and serous tumour (n = 17) samples were extracted from Affymetrix U133A GeneChip derived data that was MAS5 generated and normalized as previously described [47]. T-tests were performed to compare mean expression values of NOSE and HGSC samples using SPSS software version 16.0 (SPSS Inc., Chicago, IL, USA), where values less than 0.05 were considered significant.

*IGFBP7* expression values from primary cultures from NOSE (n = 11), HGSC samples (n = 79) and a commercially available RNA sample from a normal whole ovary (Agilent Technologies Canada Inc., Mississauga, ON, Canada) were extracted from expression data derived using a custom Ziplex® Research System gene expression array platform (Axela, Inc. Toronto, ON, Canada) that contained probes for *IGFBP7* and other genes as described elsewhere [48]. The relationship between *IGFBP7* expression values and overall or disease-free survival were evaluated using Kaplan–Meier survival curve analyses coupled to the Mantel–Cox log-rank test, and performed using SPSS software version 16.0 (SPSS Inc., Chicago, IL, USA). Values less than 0.05 were considered significant.

The *IGFBP7* expression values associated with probe set *201162_at* from 10 cytobrushings of NOSE cells (collected from surgically removed ovaries by scraping surface ovarian epithelial cells with a cotton swab) and 53 laser micro-dissected late stage HGSC samples were extracted from Affymetrix GeneChip® Human Genome U133 Plus 2.0 Array derived from a publicly available data set (E-GEOD-18520, deposited 2009-10-23 at www.ebi.ac.uk/arrayexpress/) [14] that was MAS5 generated and normalized as previously described [49]. T-tests were performed to compare mean expression values of NOSE and HGSC samples using SPSS software version 16.0 (SPSS Inc., Chicago, IL, USA), where values less than 0.05 were considered significant.

The Log2 tumour/normal ratios of *IGFBP7* expression values from 506 HGSC samples and pooled samples from eight normal fallopian tube samples was extracted from a publicly available data set deposited by The Cancer Genome Atlas (TCGA) Research Network [11] (tcga-data.nci.nih.gov/tcga/tcgaHome2.jsp) derived from using a Custom Agilent 244 K Gene Expression Microarray. Log2 values less than −1 and greater than +1 were considered significant.

### RT-PCR analysis

RT-PCR analysis was performed using cDNA prepared from 1 μg of total RNA that was extracted from tumour and EOC cell lines using Superscript III and random hexamers (Invitrogen Life Technologies, Burlington, ON, Canada), according to the manufacturer’s instructions as described previously [15,49,50]. Briefly, PCR was performed using the BIORAD DNA Engine thermal cycler using the following cycling program: 95°C for 5 minutes, 94°C for 30 seconds, 60°C for 30 seconds, 72°C for 30 seconds, and a final extension at 72°C for 5 minutes. The cycle was repeated 30 times. *IGFBP7* primers (Additional file 1) were designed using Primer3 software [51] based on reference sequence (RefSeq) *NM_001553 (IGFBP7)*, and on the genomic structure of the *IGFBP7*, as made available by the Human Genome Browser database [52]. PCR products were verified by DNA sequencing. The PCR products from RT-PCR assays were electrophoresed in 1% agarose gel and visualized by ethidium bromide staining. Primer sequences for *18S* RNA are reported in (Additional file 1).

### Western blot analysis

EOC cell lines were lysed in RIPA buffer (Sigma-Aldrich, Oakville, ON, Canada) containing the appropriate amount of protease inhibitor cocktail (Roche Diagnostics, Laval, QC, Canada). To prepare conditioned media (CM), each EOC cell line was cultured in a 100 mm diameter culture dish for 2 days in OSE complete medium, washed with PBS and incubated in serum-free medium for 2 days. CM was then collected, concentrated using Agilent Concentrator (Agilent Technologies Inc.) and filtered through 0.22 μM Millipore filter. CM was normalized to the cell number prior to loading the gel. Fifty μg of total cellular protein, CM samples or 1 ng of rIGFBP7, which was used as a positive control, were resolved by SDS-PAGE and transferred using iBlot® Gel Transfer Stacks (Life Technologies Inc. Burlington, ON, Canada) onto a nitrocellulose membrane. The membranes were blocked with 5% milk and then incubated with the appropriate antibody (Additional file 2). Proteins were visualized with ECL system (EMD Millipore, Billerica, MA, USA). Band intensity was quantified using the ImageJ open source software version IJ 1.48v (imagej.net). Each sample was normalized to the untreated sample (time = 0) and expressed as arbitrary units relative its respective control.

### Spheroid growth assays

OV90 neo^r^ and TOV112D cell lines were tested for their ability to form three-dimensional aggregates or spheroids by the hanging drop method as previously described [15,18,53]. Spheroids were incubated with CM, rIGFBP7-containing medium at the concentration of 1 or 10 ng/μl or in control complete OSE medium and formation was monitored by light microscopy over 4 days. Images were taken at ×20. The assays were preformed in triplicate.

### Wound healing assays

The ability of OV90 neo^r^ and TOV112D cell lines to migrate and fill a wound was determined using Culture-Inserts (Ibidi®, Ingersoll, Ontario, Canada), according to the manufacturer’s protocol as described previously [17]. Briefly, 50,000 cells were seeded into the outer wells of two adjacently placed Culture-Inserts in a volume of 70 ml of CM, rIGFBP7-containing medium at the concentration of 1 or 5 ng/μl or in control complete OSE medium and incubated at 37°C. Once the wells reached confluence, the Culture-Inserts were removed creating a 1-mm gap (wound). Cell migration was then monitored until the gap was filled. CM and fresh media containing rIGFBP7 were replenished every 3 days. The assay was repeated twice, in triplicate. Images were taken at ×10.

### Immunohistochemistry analysis of tissue microarrays

IGFBP7 protein expression was assessed by immunohistochemistry using a TMA containing 194 0.6 mm tumour cores of HGSC samples and 11 normal fallopian tube samples using a TMA prepared as previously described [17,18,21]. Briefly, five micron sections from the TMA were mounted on Frosted Plus slides and stained with a rabbit anti-IGFPB7 affinity purified antibody (SIGMA-ALDRICH Prestige antibody HPA002196, Additional file 2) using the Ventana Benchmark XT system (Ventana© Medical Systems, Inc., Tucson, AZ, USA). The scanned image was viewed using the Aperio ScanScope system. Two observers each scored the scanned images for the intensity of staining in the tumour epithelial cell component of each core where results were assigned as absent, low, moderate or high intensity score. The interclass correlation (average measure) between the two observer’s scores was 85%. In total 175 tumour cores and associated clinical data (Additional file 3), and 10 fallopian tubes cores were analyzed, as 19 tumour cores did not contain tumour epithelial cells.

The relationship between IGFBP7 staining intensity and either overall survival or patient disease free survival was determined by the non-parametric Mantel-Cox log rank test to compare survival distributions (SPSS software, version 16.0) and a statistic test less that 0.05 was considered significant. Survival analysis results were visualized using Kaplan-Meier survival curve analysis (SPSS software, version 16.0). Patient overall survival was defined as the time from surgery to death from ovarian cancer or last follow-up. Patient disease free survival was calculated from the time of surgery until the first progression. Clinical data on progression-free interval were defined according to level of blood CA125 and tumor size assessed by imaging. Patients known to be still alive at time of analysis were censored at time of their last follow-up.

### Sequencing

The genomic DNA for each EOC cell line (OV90, TOV21G, TOV112D, TOV81D, TOV1946, OV1946 and TOV2223) was amplified for each of the five exons of *IGFBP7* and flanking introns using previously published primer sets [54] (Additional file 1). PCR reactions were performed as described above, but using the following PCR thermal cycler conditions for each primer set: 95°C for 3 minutes, 94°C for 30 seconds, 60°C for 30 seconds, 72°C for 30 seconds, and a final extension at 72°C for 5 minutes for 35 cycles. QIAGEN HotStart Taq Plus DNA Polymerase and 5X Q-solution (Cat. No. 203603, QIAGEN Inc. Mississauga, ON, Canada) were used for the amplification of the G-C rich exon 1. PCR products were then subjected to a Sanger sequencing protocol using 3730XL DNA Analyzer systems from Applied Biosystems at the McGill University and Genome Québec Innovation Centre (gqinnovationcenter.com). Sequencing chromatograms were analysed using 4Peaks Version 1.7.2. Sequence alignment was performed using the ClustalW multiple sequence alignment platform from the European Bioinformatics Institute [55] and compared with *NM_001553 (IGFBP7)* sequence. Variants identified were compared with those reported in the Single Nucleotide Polymorphism (dbSNP) database (http://www.ncbi.nlm.nih.gov/projects/SNP/).

### Genotyping analysis of EOC cell lines

Genotyping of each EOC cell line was performed using the Infinium™ HumanHap610 genotyping BeadChip technology (Illumina, San Diego, CA, USA) as described previously (Birch et al. [56]). Genotyping and imaging of the chromosome 4 using the BeadStudio Data Analysis software (Illumina, San Diego, CA) were performed at the McGill University and Genome Quebec Innovation Centre (gqinnovationcenter.com). An image file was created and LOH was inferred by B allele frequency, where values that deviate from 0.5 (less than 0.4 and greater than 0.6) indicate allelic imbalance when reviewed for a series of adjacently mapped markers.

### DNA methylation analysis using bisulfite DNA treatment

The sodium bisulfite treatment method was used to assess methylation of *IGFBP7* using the QIAGEN EpiTect Bisulfite Kit (QIAGEN Inc., Mississauga, ON, Canada) according to the manufacturer’s instructions. Two μg of genomic DNA from each EOC cell line was used for the conversion. Methylation-specific PCR was carried out using previously published primers (Additional file 1) [37]. PCR amplification of DNA was performed using QIAGEN HotStart Taq Plus DNA Polymerase (QIAGEN Inc. Mississauga, ON, Canada) as described above under the following PCR conditions: with the methylated primers for 35 cycles at 95°C for 5 minutes, 94°C for 30 seconds, 55°C for 30 seconds, 72°C for 30 seconds, and a final extension at 72°C for 5 minutes; and with the unmethylated primers for 35 cycles at 95°C for 5 minutes, 94°C for 30 seconds, 60°C for 30 seconds, 72°C for 30 seconds, and a final extension at 72°C for 5 minutes. The PCR products were resolved in 1% gel electrophoresis.

## Results

### IGFBP7 expression in EOC cell lines

**Figure 1:**
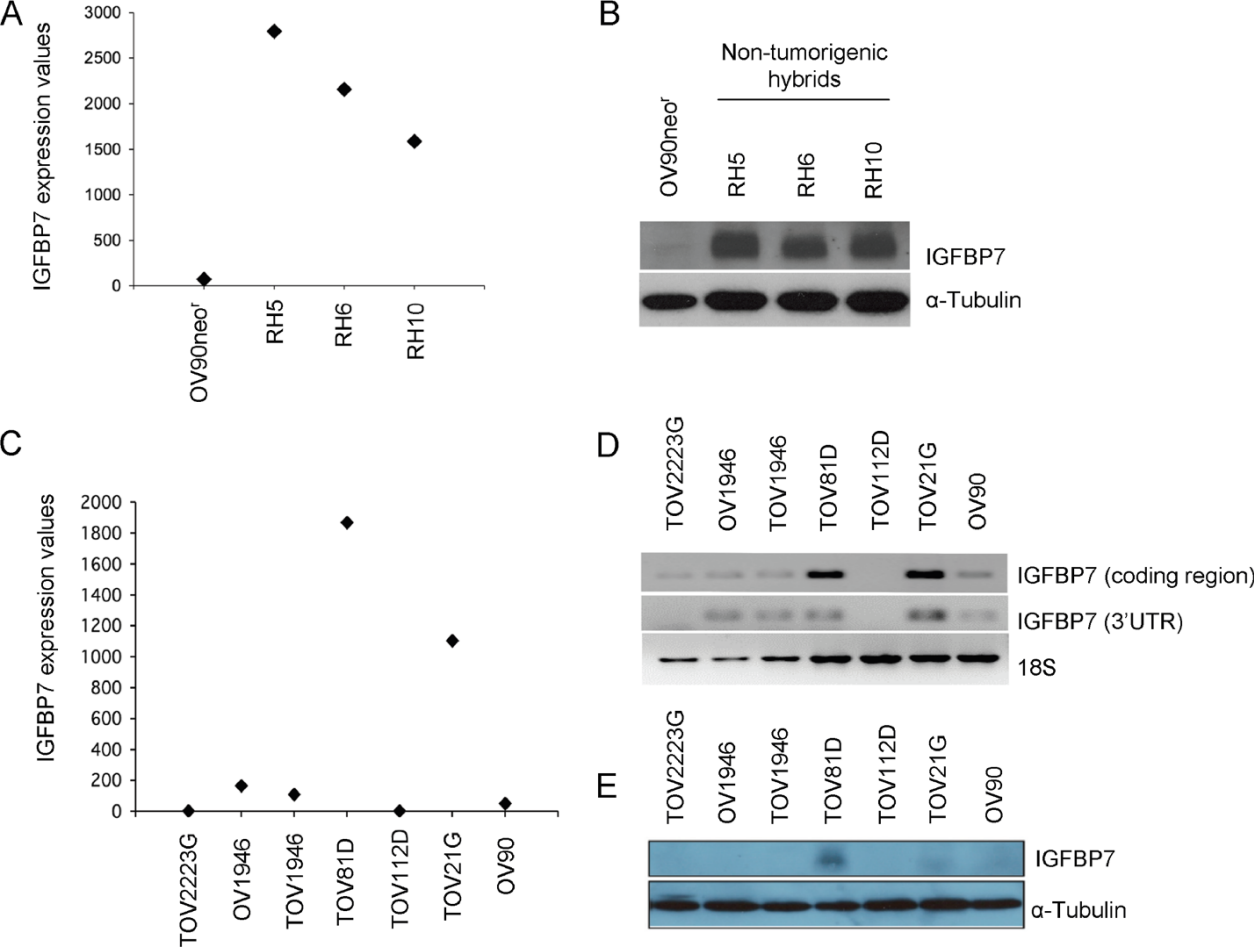
**IGFBP7 expression in ovarian cancer cell lines. A** and **C**, Gene expression microarray analysis depicting the normalized expression values of *IGFBP7* mRNA in tumourigenic parental cell line (OV90 neo^r^), three non-tumourigenic hybrids (RH5, RH6, RH10) and in six additional EOC cell lines, using Affymetrix U133Plus2 microarray platform in **A**, and the custom Ziplex® Research System gene expression array platform in **C**. **B** and **E**, IGFBP7 intracellular protein level in EOC cell lines and the three non-tumourigenic hybrids was analysed by western blot analysis. α-tubulin was used as a loading control. **D**, Semi-quantitative RT-PCR analysis of IGFBP7 in seven EOC cell lines using 2 different couples of primers. The expression of *18S* is shown as an internal control.

Our group previously investigated the transcriptome reprogramming that occurred in three genetically modified OV90 clones (RH5, RH6 and RH10), that were rendered non-tumourigenic as a consequence of an unique gene complementation assay involving the transfer of normal chromosome 3 in a study aimed at identifying chromosome 3 tumour suppressor genes [15,16,18,20]. Among the genes dysregulated as a consequence of tumour suppression, *IGFBP7* exhibited a strong upregulation as measured by Affymetrix microarray analysis (Figure 1A). Western blot analysis showed corresponding high levels of IGFBP7 protein in the hybrids relative to the parental OV90 cell line (Figure 1B). *IGFBP7* expression was then investigated in six other independently derived EOC cell lines established as long-term passages from chemotherapy naïve EOC patients [19,46]. As shown by custom gene expression microarray analysis, the highest levels of expression were observed in TOV81D and TOV21G relative to all other cell lines tested (Figure 1C). The observations were consistent with RT-PCR results (Figure 1D). TOV81D also expressed the highest level of protein by western blot analysis (Figure 1E). These findings are interesting in light of the observation that TOV81D is unable to grow without solid support *in vitro* and does not form tumours at peritoneal sites in nude mice [19]. TOV81D cell line was derived from a low grade papillary serous adenocarcinoma specimen from a patient who survived seven years post surgery, suggestive of a less aggressive form of the disease [19] (Table 1).

**Table 1:** **IGFBP7 expression, mutation and genotyping analyses relative to EOC cell lines features and growth characteristics**

Cell lines	Histopathology	Grade	Stage	Source	Age	*TP53*mutation status*	Growth in soft agar*	Spheroid formation*	Tumour formation in nude mouse*		*IGFBP7*mutation	Genotype*IGFBP7*locus	*IGFBP7*methylation	*IGFBP7*transcript expression	IGFBP7 protein detection in total cell lysate
									Subcutaneous	Intraperitoneal					
TOV2223G	Serous papillary cystadenocarcinoma	3	III-C	Tumor	89	Positive	Yes	No	No	No	No	LOH	Yes	Low	No
OV1946	Serous papillary cystadenocarcinoma	3	III-C	Ascites	75	Positive	Yes	Semi-compact	No	Yes	No	LOH	No	Low	No
TOV1946	Serous papillary cystadenocarcinoma	3	III-C	Tumor	75	Positive	Yes	Aggregate	No	Yes	No	LOH	No	Low	No
TOV81D	Serous papillary adenocarcinoma	1-2	III-C	Tumor	66	Negative	No	No	No	No	No	HET	No	High	High
TOV112D	Endometrioid carcinoma	3	III-C	Tumor	42	Positive	Yes	Yes	Yes	Yes	No	LOH	Yes	No	No
TOV21G	Clear cell carcinoma	3	III	Tumor	62	Negative	Yes	Yes	Yes	Yes	No	HET	No	High	Low
OV90	Adenocarcinoma	3	III-C	Ascites	64	Positive	Yes	Yes	Yes	Yes	No	AI	No	Low	No

*From Provencher et al. [19]; Ouellet et al. [46]; Cody et al [15]. n/d: not determined, LOH: Loss of heterozygosity, HET: heterozygote, AI: allelic imbalance.

### IGFBP7 expression profiles in ovarian cancer samples and reference normal tissues

**Figure 2:**
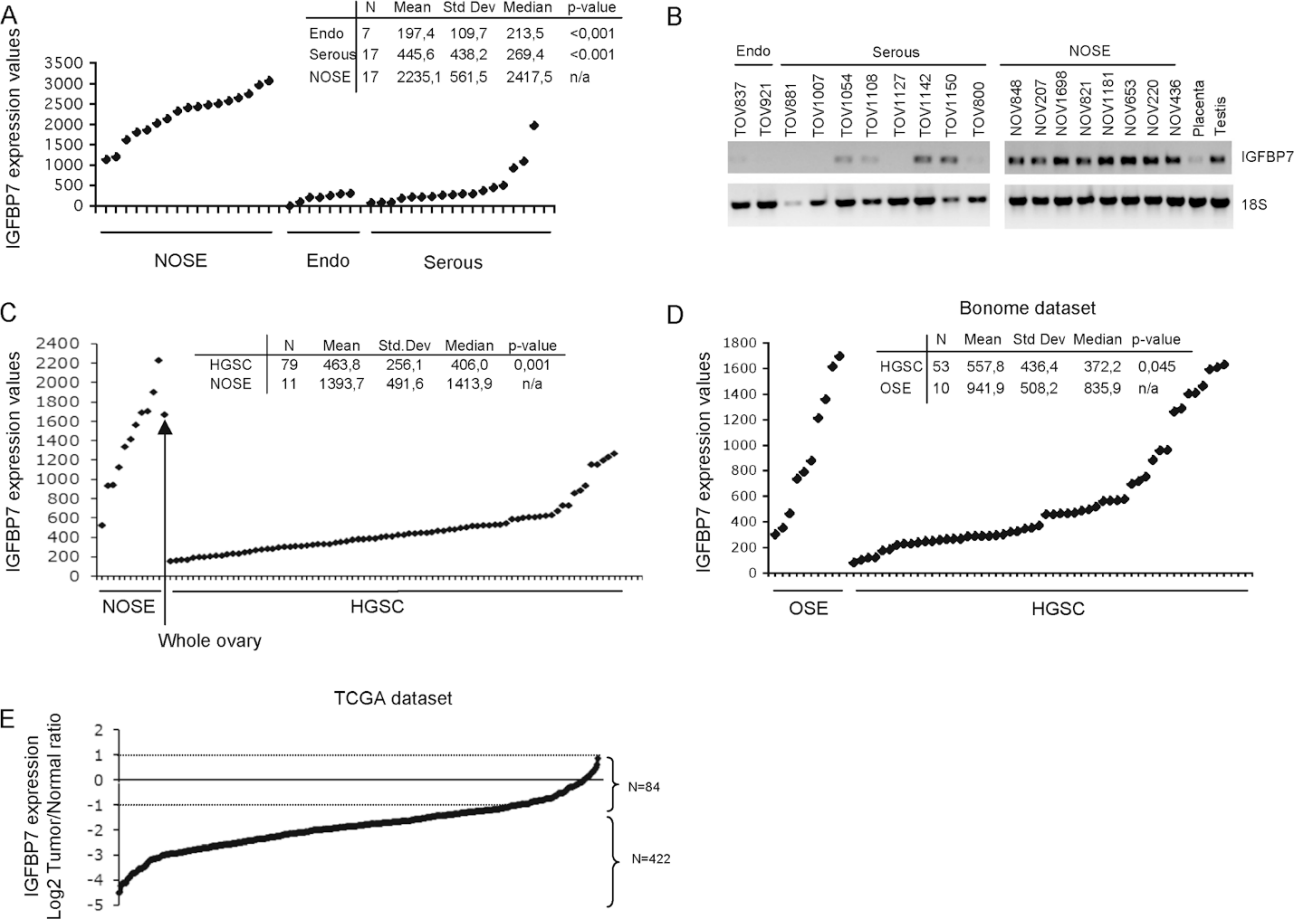
***IGFBP7*expression profile in normal and malignant ovarian cancer samples. A**, Affymetrix U133A gene expression microarray analysis of *IGFBP7* in NOSE (n = 17) and TOV samples: endometrioid (Endo) (n = 7) and serous subtypes (n = 17). **B**, Semi-quantitative RT-PCR analysis of *IGFBP7* in NOSE and TOV samples. *18S* was used as an internal control. **C**, *IGFBP7* expression in NOSE samples (n = 11), normal whole ovary and HGSC samples (n = 79) as assayed by Ziplex Research System expression array. **D**, *IGFBP7* expression from the Bonome dataset assessed by Affymetrix U133 Plus 2.0 in cytobrushing of OSE (n = 10) and HGSC subjected to laser microdissection (n = 53). **E**, Log2 tumour/normal ratios of expression values for *IGFBP7* gene assessed using Custom Agilent 244 K Gene Expression Microarray in 506 HGSC samples relative to the expression values derived from adjacent normal tissue or case matched peripheral blood lymphocytes (TCGA dataset).

We investigated *IGFBP7* expression in tumour samples and reference surface normal epithelial cells from samples derived from our tissue banks (Additional file 4). Our analysis of control samples was limited to RNA from NOSE, as fallopian tube samples, also proposed as tissues of origin for HGSC [57,58], were not available in our tissue bank. Overall gene expression was significantly underexpressed in HGSC samples relative to primary cultures of NOSE samples using Affymetrix (p < 0.001) and the tailored Ziplex Research Systems gene expression assay (p < 0.001) (Figure 2A and C). Low expression was also observed in the seven HGEC samples relative to primary cultures of NOSE samples (Figure 2A). Our analysis of a limited number of samples by RT-PCR showed that four of eight serous samples exhibited evidence of *IGFBP7* expression, where only two tumour samples showed robust expression (Figure 2B). All 79 HGSC samples exhibited *IGFBP7* expression at levels lower than that found in the reference whole ovary sample (Figure 2C). It is estimated that less than 10% of whole ovary contains surface epithelial cells, one of the proposed tissues of origin of EOC, while stromal cells make up the predominant cell type [58-60]. Clinical information was available for all of the 79 HGSC samples investigated. We found no significant relationship between *IGFBP7* expression and overall survival or progression-free survival in this sample set (data not shown; Chi = 0.712 and p = 0.399 for overall survival and Chi = 0.275 and p = 0.6 for progression free survival).

We also investigated the *IGFBP7* expression profiles from publicly available data. The results showed significant underexpression in HGSC samples in the independently derived Affymetrix U133 Plus 2 data set (Bonome dataset, p = 0.045) [14] (Figure 2D). Similar findings were also observed by the TCGA group where 422 (83.4%) samples exhibited greater than two-fold differences gene expression values relative to normal reference tissue, where a custom made Agilent gene expression array system was applied (Figure 2E). All of these findings were notable for comparison of reference tissue used in the respective analyses. The results where brushing of NOSE were used in the Bonome dataset, and adjacent normal tissue or peripheral lymphocytes used by the TCGA group were comparable to that observed in our assays where primary cultures of NOSE were used (Figure 2A and C).

### IGFBP7 protein expression and clinical parameters

**Figure 3:**
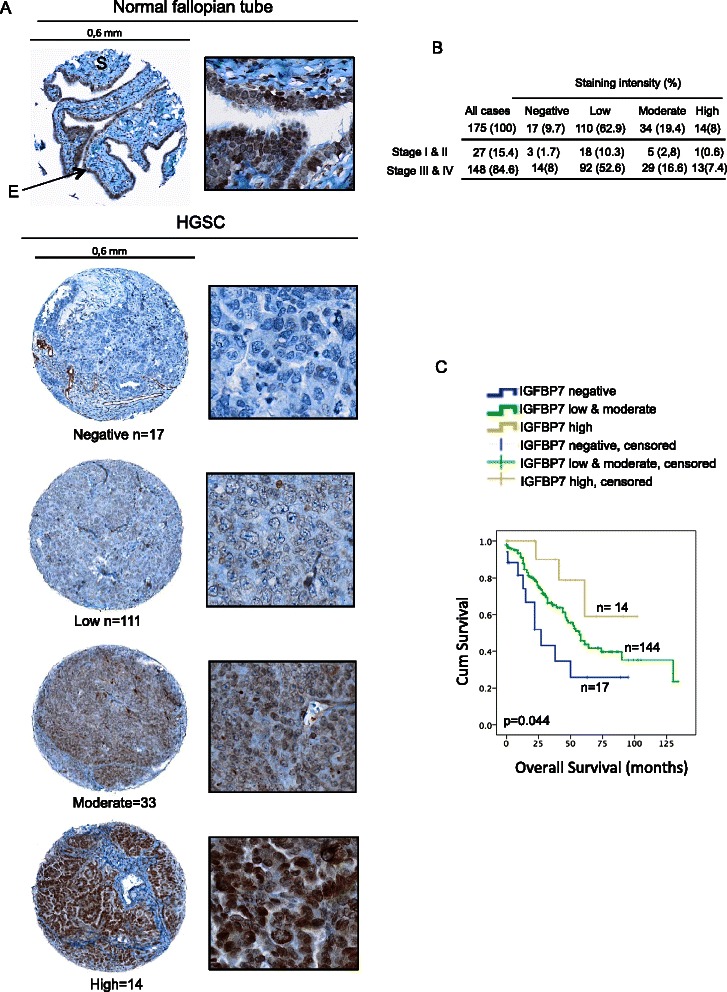
**IGFBP7 protein expression in high-grade serous ovarian carcinomas and normal fallopian tube samples. A**, Examples of IGFBP7 staining patterns in a representative cores from a normal fallopian tube tissue sample (up panel, magnification × 20), and in cores from HGSC samples (bottom panel, magnification × 20) showing negative, low, moderate and high staining patterns. ‘E’ indicates epithelial cells and ‘S’ indicates stromal cells. Immunohistochemistry images were obtained from the OlyVIA image viewer (Olympus America Inc.). **B**, Percentages refer to the proportion of the 175 HGSC samples showing the staining patterns of protein expression. **C**, Kaplan–Meier survival curve analysis of HGSC cases for overall survival of patients whose tumours showing the following staining patterns for IGFBP7 protein: negative (n = 17), low and moderate combined (n = 144), and high (n = 14). All p-values were derived from log-rank tests.

IHC analysis was performed to characterize IGFBP7 protein expression in fallopian tube as the expression profile in human tissues purported to be the origins of HGSCs have not previously been described in research, and whole ovary samples with intact NOSE were not available on the tissue microarray (TMA). Though staining was evident in both the epithelial and stromal cells of this tissue, it was more evident in the epithelial cells lining the surface of fallopian tubes (Figure 3A). Though variable staining intensity was observed across the 10 normal fallopian tube tissues examined by IHC of the TMA, nine of 10 samples displayed positive staining (data not shown). Our staining patterns are consistent with those observed in the Human Protein Atlas, which applied the same antibody to human tissues (http://www.proteinatlas.org/ENSG00000163453-IGFBP7/tissue).

IGFBP7 protein expression in HGSC was also investigated by IHC analysis on the same TMA containing 194 cores from HGSC. The analysis includes results from 175 cores as 19 cores lacked sufficient tumour epithelial cell components for evaluation. Though staining was observed in both epithelial and stromal cells of the tumour, only staining from the tumour epithelial cells was investigated in further analyses. Both cytoplasmic and nuclear staining was observed in the tumour tissues (Figure 3A), as it is also evident from the results of the Human Protein Atlas (http://www.proteinatlas.org/ENSG00000163453-IGFBP7/tissue). Figure 3 contains examples of the staining pattern in epithelial cells in the 175 HGSC cores, which ranged from absent (9.7%), low (62.9%), moderate (19.4%) and high (8%) intensity. Thus, the majority of the tumour samples (72.6%) expressed either absent or low levels of IGFBP7 protein (Figure 3B) and these results overall are consistent with low levels of gene expression.

Clinical data was available for the 175 cases examined for IGFBP7 immunostaining (Additional file 3). Our analyses showed that there was no statistically significant difference in the distribution of staining intensity patterns and disease stage (data not shown). The relationship between IGFBP7 immunostaining and overall or disease-free survival was evaluated using Kaplan–Meier survival curve analyses. The analyses were performed using all possible combinations based on staining patterns grouped according to absent, low, moderate and high staining levels (Figure 3C and Additional file 5) No significant relationship between IGFBP7 protein expression and either overall (log rank = 6.68, p = 0.083) or disease-free survival (log rank = 1.68, p = 0.641) was observed when comparisons were made in consideration of these four staining categories (Additional file 5). However, there was a significant association between prolonged overall survival and the presence of IGFBP7 protein when the cases with no staining are compared with those with low and moderate staining combined and those of high staining (p < 0.044) (Figure 3C) or when cases were examined for the absence or presence of staining (p < 0.040) (Additional file 5).

### Mechanisms of inactivation of IGFBP7 expression in EOC cell lines

We investigated genetic and epigenetic mechanisms of inactivation of *IGFBP7* expression as independent studies have demonstrated such classical mechanisms may abrogate *IGFBP7* function in various cancer types [25,26,37,40,42,43]. We focused our analysis on the EOC cell lines that were initially investigated for gene and protein expression (Figure 1). Genotyping analyses using whole genome SNP array showed LOH of the *IGFBP7* locus at 4q12 in TOV112D, TOV1946, OV1946, and TOV2223G; allelic imbalance in OV90; and no evidence of genomic anomalies in TOV21G and TOV81D (Additional file 6). Notable is that *IGFBP7* expression was clearly evident in TOV21G and TOV81D, the only cell lines exhibiting no evidence of genomic anomalies at 4q12 locus by SNP array analysis.

A predicted CpG island overlapping the first exon of *IGFBP7* was investigated for methylation analysis [52] as evidence for alteration of methylation status has been demonstrated in the analysis of a variety of other cancer types [25,26,37,40,42,43]. Methylation analysis was performed using a combination of Bisulfite DNA treatment on ovarian cell line DNA and methylation-specific PCR. Evidence of methylation was observed for TOV112D and TOV2223G cell lines, which were derived form a low- and high-grade serous carcinoma respectively (data not shown). Notable is that neither TOV112D nor TOV2223G exhibited evidence of *IGFBP7* gene and protein expression (Figure 1).

### IGFBP7 protein and effect on in vitro growth phenotypes of EOC cell lines

**Figure 4:**
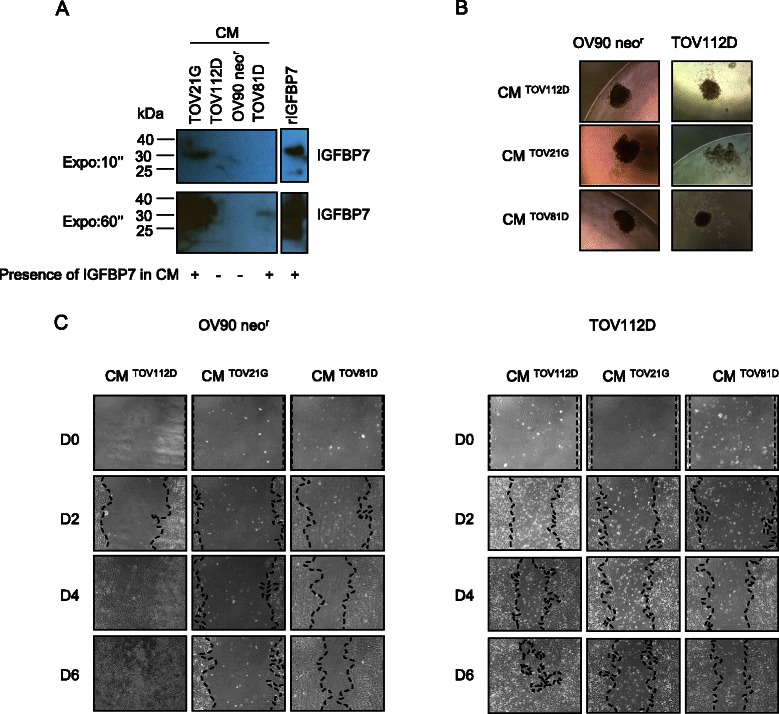
**Effects of IGFBP7-containing conditioned media on EOC cell lines. A**, Immunoblot analysis monitoring IGFBP7 protein levels in the CM from EOC cell lines. Recombinant IGFBP7 protein was used as a positive control and two different exposures are shown (10 and 60 seconds). **B**, Spheroid formation assay of OV90 neo^r^ and TOV112D cell lines cultured in IGFBP7-containing CM (TOV21G and TOV81D) or control CM (TOV112D). Images are shown at × 20 magnification. **C**, Wound healing assay with OV90 neo^r^ and TOV112D cultured in IGFBP7-containing CM (TOV21G and TOV81D) or control CM (TOV112D) observed over a period of 6 days. Images are shown at × 10 magnification.

We assessed the effect on the growth phenotypes of EOC cell lines of either conditioned media (CM) derived from IGFBP7-expressing cells, or commercially available human recombinant IGFBP7 (rIGFBP7) protein, as IGFBP7 is also a secreted protein [61]. As shown in Figure 4A, western blot analysis of CM derived from cultures of TOV81D and TOV21G cell lines provided evidence of secreted IGFBP7 protein, while CM derived from TOV112D and OV90 neo^r^ showed absence of IGFBP7. These results are consistent with our previous gene and protein expression profiles of the EOC cell lines (Figure 1).

We focused our further analyses on TOV112D and OV90 neo^r^ cell lines, as they expressed no detectable levels of IGFBP7 protein (Figures 1 and 4A). Cell viability assays showed that the growth rate of OV90 neo^r^ and TOV122D was not affected by the presence of IGFBP7-containing CM from either TOV81D or TOV21G (data not shown). We previously reported the ability of both OV90 neo^r^ and TOV112D to form a compact spheroid in hanging drop cultures [19]. This ability did not seem to be affected by IGFBP7-containing CM treatments, with the exception of TOV112D, where the cell aggregates formed appeared less compact when treated with the CM from TOV21G, compared to the CM from TOV81D and the control CM from TOV112D (Figure 4B). We next assessed the effect of IGFBP7-containing CM on cell migration using a “wound-healing” assay. As shown in Figure 4C, the rate at which both OV90 neo^r^ and TOV112D cells migrated into and filled the gap (wound) were noticeably affected when cultured with IGFBP7-containing CM as compared to control CM.

**Figure 5:**
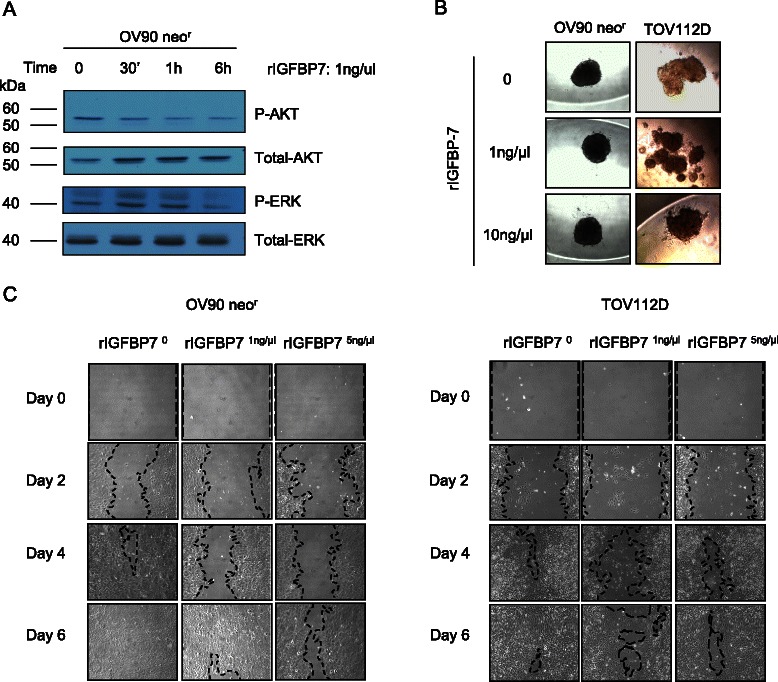
**Effects of IGFBP7 recombinant protein on EOC cell lines. A**, Western blot analysis showing an alteration of the phophorylation status of ERK and AKT proteins after rIGFBP7 treatment of 1 ng/μl of OV90 neo^r^ cell line as determined at different time points following exposure. Total AKT and total ERK served as a loading control. **B**, Spheroid formation assay with OV90 neo^r^ and TOV112D cell lines in the presence or absence of rIGFBP7 (1 and 10 ng/μl). Images are shown at ×20 magnification. **C**, wound healing assay of OV90 neo^r^ and TOV112D in the presence or absence of rIGFBP7 (1 and 5 ng/μl) observed over a period of 6 days. Images are shown at × 10 magnification.

To verify if the inhibition of the migratory effect was due to the presence of IGFBP7 in CM, we repeated the growth assays using a recombinant IGFBP7 (rIGFBP7) protein. Independent studies have shown that IGFBP7 mediates its biological effect via the inhibition of the MEK-ERK pathway [24,62] and AKT pathway [63]. Therefore we began our investigation of the activity of the r-IGFPB7 protein by assaying the level of ERK and AKT protein phosphorylation by western blot analysis in total cellular lysate from OV90 cell line exposed or not to r-IGFBP7 treatment. As shown in Figure 5A, phosphorylation of AKT and ERK decreased in OV90 neo^r^ cells after 30 minutes and 6 hours of exposure to rIGFBP7 respectively (Additional file 7). This observation is consistent with independent reports showing an inverse correlation of AKT and ERK phosphorylation with IGFBP7 treatment or gene expression [23,24,34,63,64]. Cell viability assays showed no significant difference in the growth rate of OV90 neo^r^ and TOV122D when cultured in the presence of rIGFBP7 (data not shown). Exposure to rIGFBP7 protein did not affect the ability of OV90 neo^r^ to form aggregates in hanging drop cultures (Figure 5B). Interestingly, TOV112D treated with 1 ng of IGFBP7 was unable to form a single large compact spheroid in contrast that observed when exposed to 10 ng of IGFBP7, and this observation was reproducible. The rate at which OV90 neo^r^ and TOV112D cells migrated into and filled the gap (wound) were noticeably affected in cultures exposed to rIGFBP7 protein, which is consistent with the effects of IGFBP7-containing CM on the same cell lines.

## Discussion

In this study we have shown for the first time significant *IGFBP7* under-expression in HGSC samples relative to reference NOSE cells. These results are intriguing in light of our observation of IGFBP7 protein expression in epithelial cells of distal fimbrae of the fallopian tube, as both NOSE and fallopian tubes have been proposed as the progenitor cell type for HGSC [58,65,66]. Although it is difficult to directly compare gene expression with protein expression using IHC results, only about 8% of HGSC samples exhibited strong staining of epithelial tumour cells by IHC analysis of a TMA containing tumour cores as compared with about 72.6% of samples which showed absent or low staining levels. This suggests the possibility that IGFBP7 protein levels are indeed low or absent in the vast number of epithelial tumour cells of HGSCs.

We observed a significant positive association between IGFBP7 staining intensity in the epithelial cells of HGSC samples and overall survival. In our TMA analyses, the samples with no IGFBP7 staining were from patients that exhibited the poorest outcome, suggesting a role of IGFBP7 in molecular pathways associated with favourable outcome in HGSC. To our knowledge, our study is the first showing a significant correlation between expression of the protein and a more favourable patient outcome in HGSC. An association between low IGFBP7 expression and poor outcome was also observed in pancreatic ductal, breast and hepatocellular adenocarcinoma [26,35-38,40,42,44,45]. These observations suggest a role of IGFBP7 in molecular pathways associated with favourable outcome in HGSC and other cancer types. In contrast, IGFBP7 expression has also been associated with poorest outcome of patient with oesophagial adenocarcinoma [67] or colorectal cancer [68]. It has become increasingly apparent that the levels of IGFBP7 differ in different cancer types, where relatively higher levels of expression have been described in gliobastomas multiforme [63], oesophageal squamous cell [69], and colorectal carcinoma [70]. The differences in protein expression might reflect a context dependent function of IGFBP7, although this requires further exploration.

Our findings with HGSC tissues and normal cells are largely consistent with the gene expression in EOC cell lines. With the exception of TOV81D and TOV21G, our EOC cell lines exhibited low levels or the absence of both gene and protein expression. The *IGFBP7* expression in TOV81D is interesting as this cell line is not tumourigenic in immunocompromised mouse tumour xenograft models and was derived from a sample from a patient having an unusually prolonged overall survival which was in excess of 7 years [19]. TOV21G was derived from a patient with a clear cell carcinoma, a disease which exhibits distinct clinical course that differs from HGSC, HGEC and undifferentiated adenocarcinomas, cancer types from which the other EOC cell lines were derived (see Table 1) [19]. Moreover, TOV21G is an unusually rare case of EOC in that it shows evidence of methylated alleles in *MLH1*, a mismatch repair gene [56] and thus it is possible that molecular pathways that implicate *IGFBP7* differ in this cell line as compared to other EOC cell lines studied. Consistent with this notion is a recent report showing that TOV21G exhibited a ‘hypermutator’ genotype along with a low alteration of copy number alteration, a phenotype distinct from the other 46 EOC cell lines examined in the study and from HGSC [71]. Thus, our results, suggest that underexpression is associated with tumourigenicity and that expression of *IGFBP7* may be important for tumour suppressor pathway phenotype.

The absence of *IGFBP7* expression in OV90, TOV112D, TOV2223, TOV1946 and OV1946 is also interesting given the observation of LOH or allelic imbalance of the *IGFBP7* locus at 4q12 in these EOC cell lines as compared to TOV81D and TOV21G. These findings are consistent with a high frequency (in greater than 50% of the tumours) of LOH and copy number loss involving the 4q arm as deduced by genome wide genotyping assays in our analysis of 79 HGSC samples that were examined in the present study and reported previously by our group [10], and in the analysis of 489 HGSC samples that were examined by the TCGA group [11]. Somatic mutation and epigenetic analyses of our cell lines, however, only demonstrated the possibility of promoter epigenetic silencing occurs in TOV2223G and TOV112D. These observations are consistent with recent reports from the TCGA analysis of HGSCs [11], where there was no evidence of *IGFBP7* somatic mutations by exome sequencing analysis (n = 316 samples) nor for epigenetic silencing by CpG methylation array analyses (n = 489 samples) [11]. *IGFBP7* intragenic mutations were also not found in relation to down-regulation in breast and colorectal cancers [54], although there is evidence of epigenetic silencing occurring in other types of cancer cell lines [24-26,41]. Thus, when taken together, other mechanisms may be involved in regulating *IGFBP7* expression in HGSC.

The absence or low levels of *IGFBP7* expression observed in HGSC samples is consistent with our observation that *IGFBP7* was among the list of genes up-regulated (reprogrammed) in the context of suppressing tumourigenic potential in our chromosome 3 complementation assays involving OV90 [15]. *IGFBP7* has been proposed as a candidate tumour suppressor gene as suggested by experiments demonstrating suppression of tumourigenicity in murine lung, prostate and colorectal, breast and skin cancers xenograft models with rIGFBP7 [25,26,35,62,72]. Thus, our observations that rIGFBP7 affects the migration rate of cells in *in vitro* wound healing assays of EOC cell lines is intriguing. However, rIGFBP7 appears to have no significant impact on cell proliferation, viability or spheroid formation (though there was a modest effect of spheroid formation with TOV112D). Wajapyee et al. reported that the induction of the apoptotic process by *IGFBP7* largely occurred in NCI60 human cancer cell lines that harboured an activated *BRAF* or *RAS* mutation. Among the six ovarian cancer cell lines treated with rIGFBP7 in that study, only OVCAR5, which harbours an activated *RAS* mutation, exhibited susceptibility to rIGFBP7 as measured by the percentage of apopototic cells 24 hours after treatment [62]. Neither OV90 nor TOV112D EOC cell lines investigated in our study harbour activating *BRAF* or *RAS* mutations [19,46]. Moreover mutations in these genes are not a feature of HGSCs [7,11]. In breast cancer, IGFBP7 treatment inhibited cell growth and induced apoptosis and senescence, *in vitro* and *in vivo*, only in cell lines that were tested negative for HER2/neu, estrogen and progesterone [72]. In the same report, IGFBP7 effects were associated with strong activation of the p38 MAPK pathway and both p53 and p21^cip1^ were up-regulated implicating known senescence pathways involving these proteins [72]. In light of these observations, it is therefore interesting that both OV90 and TOV112D harbour somatic *TP53* mutations, a feature of over 90% of HGSCs [8-10,73]. Thus rIGFBP7 may have interacted with factors in alternative pathways to result in the effects observed with OV90 and TOV112D in wound healing assays.

In our study, rIGFBP7 protein inhibited the phosphorylation of both ERK and AKT protein in OV90 cell line, which is consistent with reports of similar assays with different human cell lines types [23,24,34,63,64]. Biochemical analyses have revealed that IGFBP7 interacts with activin, a member of the TGFβ superfamily of signalling proteins [29], and that IGFBP7 can be activated by TGFβ proteins and retinoic acid [74-76]. A recent study revealed that IGFBP7 acts as an IGF1/2 antagonist by directly binding to IGF-1 receptor (IGF1R) and hindering its activation and internalization, which results in blocking downstream phosphatidylinositol 3-kinase (PI3K)-AKT signalling and thereby inhibiting protein synthesis, cell growth and survival [34]. These observations are intriguing given the high frequency of AKT signalling pathway activation in HGSC [77-79], whereby the TCGA study identified alteration in the PI3K/AKT and RAS pathways in approximately 45% of HGSC [11].

Recent studies have reported an important role of IGFBP7 in therapy sensitization in different types of invasive cancers [80-82]. In acute myeloid leukemia cells, IGFBP7 cooperates with chemotherapy to induce cell cycle arrest and apoptosis and this mechanism is independent of ERK and AKT activation [82]. Interestingly, IGFBP7 has been identified with IGFBP4 in the secretome of mesenchymal stem cells and promotes their senescence [83]. While the mechanism has not been elucidated it has been proposed that this interaction participates in the inhibition of stem cell renewal and cancer development [83].

## Conclusion

IGFBP7 is underexpressed in the majority of HGSCs, and protein expression is correlated to a prolonged overall survival. Given the known tumour suppressor activity of IGFBP7 in several cancer types, understanding the importance of maintaining low/absent levels of IGFBP7 in HGSC is warranted to further elucidate the role of this protein in the development and progression of this disease.

## Additional files

Additional file 1:
**Primers used in RT-PCR and sequencing assays.**

Additional file 2:
**Antibodies used in western blot and immunocytochemistry analyses.**

Additional file 3:
**Description of HGSC cohort used in TMA.**

Additional file 4:
**Features of ovarian tumour samples used in U133A Affymetrix gene chip and RT-PCR analyses.**

Additional file 5: **Kaplan-Meier survival curve analyses of IGFBP7 staining intensity in HGSCs.** A, Kaplan–Meier survival curve analysis of HGSC cases for overall survival of patients whose tumours showed negative (n = 17), low (n = 110), moderate (n = 34) and high (n = 14) IGFBP7 staining. B, Kaplan–Meier survival curve analysis of HGSC cases for overall survival of patients whose tumours showed negative (n = 17), and positive (n = 158) IGFBP7 staining. All p-values were derived from log-rank tests.
Additional file 6: **SNP array imaging for chromosome 4 of the EOC cell lines.** The top plot of each figure shows B allele frequencies for each chromosome 4 SNP marker aligned to its chromosomal position. The bottom plot of each figure contains the log R ratio, which provides an indication of the copy number for each SNP marker aligned to its chromosomal position and a red bar indicates IGFBP7 locus. Note the complete LOH of the entire chromosome 4 for TOV2223G, OV1946, TOV1946 and TOV112D. OV90 exhibits an allelic imbalance for the whole chromosome.
Additional file 7: **Quantification of western blots results of P-AKT and P-ERK in OV90neo**^**r**^**cells exposed to rIGFBP7.** Band intensities were quantified using ImageJ software, normalized to untreated samples and expressed as arbitrary units relative to controls.

## Abbreviations

IGFBP7: Insulin-like growth factor binding protein 7
rIGFBP7: Recombinant protein of IGFBP7
HGSC: High-grade serous ovarian carcinomas
EOC: Epithelial ovarian cancer
NOSE: Normal ovarian surface epithelial cells
TCGA: The Cancer Genome Atlas
CM: Conditioned media
IHC: Immunohistochemistry
TMA: Tissue microarray
OS: Overall survival
PFS: Progression-free survival
LOH: Loss of heterozygosity

## References

[1] CCS (2012). Canadian Cancer Statistic 2012.

[2] ACS (2012). Cancer Facts & Figures 2012.

[3] Vaughan S, Coward JI, Bast RC, Berchuck A, Berek JS, Brenton JD (2011). Rethinking ovarian cancer: recommendations for improving outcomes. Nat Rev Cancer.

[4] (PDQ®) PDQ (2013). Physician Data Query; Ovarian Epithelial Cancer Treatment (PDQ®).

[5] Rosen DG, Yang G, Liu G, Mercado-Uribe I, Chang B, Xiao XS (2009). Ovarian cancer: pathology, biology, and disease models. Front Biosci.

[6] Lynch HT, Casey MJ, Snyder CL, Bewtra C, Lynch JF, Butts M (2009). Hereditary ovarian carcinoma: heterogeneity, molecular genetics, pathology, and management. Mol Oncol.

[7] Berns EM, Bowtell DD (2012). The changing view of high-grade serous ovarian cancer. Cancer Res.

[8] Ahmed AA, Etemadmoghadam D, Temple J, Lynch AG, Riad M, Sharma R (2010). Driver mutations in TP53 are ubiquitous in high grade serous carcinoma of the ovary. J Pathol.

[9] Gorringe KL, Campbell IG (2009). Large-scale genomic analysis of ovarian carcinomas. Mol Oncol.

[10] Wojnarowicz PM, Oros KK, Quinn MCJ, Arcand SL, Gambaro K, Madore J (2012). The genomic landscape of TP53 and p53 annotated high grade ovarian serous carcinomas from a defined founder population associated with patient outcome. PLoS One.

[11] TCGA (2011). Integrated genomic analyses of ovarian carcinoma. Nature.

[12] Vang R, Shih Ie M, Kurman RJ (2009). Ovarian low-grade and high-grade serous carcinoma: pathogenesis, clinicopathologic and molecular biologic features, and diagnostic problems. Adv Anat Pathol.

[13] Schwartz DR, Kardia SL, Shedden KA, Kuick R, Michailidis G, Taylor JM (2002). Gene expression in ovarian cancer reflects both morphology and biological behavior, distinguishing clear cell from other poor-prognosis ovarian carcinomas. Cancer Res.

[14] Bonome T, Lee JY, Park DC, Radonovich M, Pise-Masison C, Brady J (2005). Expression profiling of serous low malignant potential, low-grade, and high-grade tumors of the ovary. Cancer Res.

[15] Cody NA, Ouellet V, Manderson EN, Quinn MC, Filali-Mouhim A, Tellis P (2007). Transfer of chromosome 3 fragments suppresses tumorigenicity of an ovarian cancer cell line monoallelic for chromosome 3p. Oncogene.

[16] Cody NA, Shen Z, Ripeau JS, Provencher DM, Mes-Masson AM, Chevrette M (2009). Characterization of the 3p12.3-pcen region associated with tumor suppression in a novel ovarian cancer cell line model genetically modified by chromosome 3 fragment transfer. Mol Carcinog.

[17] Wojnarowicz P, Gambaro K, de Ladurantaye M, Quinn MC, Provencher D, Mes-Masson AM (2012). Overexpressing the CCL2 chemokine in an epithelial ovarian cancer cell line results in latency of in vivo tumourigenicity. Oncogenesis.

[18] Gambaro K, Quinn MC, Wojnarowicz PM, Arcand SL, de Ladurantaye M, Barres V (2013). VGLL3 expression is associated with a tumor suppressor phenotype in epithelial ovarian cancer. Mol Oncol.

[19] Provencher DM, Lounis H, Champoux L, Tetrault M, Manderson EN, Wang JC (2000). Characterization of four novel epithelial ovarian cancer cell lines. In Vitro Cell Dev Biol Anim.

[20] Quinn MC, Filali-Mouhim A, Provencher DM, Mes-Masson AM, Tonin PN (2009). Reprogramming of the transcriptome in a novel chromosome 3 transfer tumor suppressor ovarian cancer cell line model affected molecular networks that are characteristic of ovarian cancer. Mol Carcinog.

[21] Quinn MC, Wojnarowicz PM, Pickett A, Provencher DM, Mes-Masson AM, Davis EC (2013). FKBP10/FKBP65 expression in high-grade ovarian serous carcinoma and its association with patient outcome. Int J Oncol.

[22] Burger AM, Leyland-Jones B, Banerjee K, Spyropoulos DD, Seth AK (2005). Essential roles of IGFBP-3 and IGFBP-rP1 in breast cancer. Eur J Cancer.

[23] Wajapeyee N, Serra RW, Zhu X, Mahalingam M, Green MR (2008). Oncogenic BRAF induces senescence and apoptosis through pathways mediated by the secreted protein IGFBP7. Cell.

[24] Vizioli MG, Sensi M, Miranda C, Cleris L, Formelli F, Anania MC (2010). IGFBP7: an oncosuppressor gene in thyroid carcinogenesis. Oncogene.

[25] Ruan WJ, Lin J, Xu EP, Xu FY, Ma Y, Deng H (2006). IGFBP7 plays a potential tumor suppressor role against colorectal carcinogenesis with its expression associated with DNA hypomethylation of exon 1. J Zhejiang Univ Sci B.

[26] Chen Y, Pacyna-Gengelbach M, Ye F, Knosel T, Lund P, Deutschmann N (2007). Insulin-like growth factor binding protein-related protein 1 (IGFBP-rP1) has potential tumour-suppressive activity in human lung cancer. J Pathol.

[27] Chen D, Yoo BK, Santhekadur PK, Gredler R, Bhutia SK, Das SK (2011). Insulin-like growth factor-binding protein-7 functions as a potential tumor suppressor in hepatocellular carcinoma. Clin Cancer Res.

[28] Firth SM, Baxter RC (2002). Cellular actions of the insulin-like growth factor binding proteins. Endocr Rev.

[29] Kato MV (2000). A secreted tumor-suppressor, mac25, with activin-binding activity. Mol Med.

[30] Yamanaka Y, Wilson EM, Rosenfeld RG, Oh Y (1997). Inhibition of insulin receptor activation by insulin-like growth factor binding proteins. J Biol Chem.

[31] Nousbeck J, Sarig O, Avidan N, Indelman M, Bergman R, Ramon M (2010). Insulin-like growth factor-binding protein 7 regulates keratinocyte proliferation, differentiation and apoptosis. J Invest Dermatol.

[32] Walker GE, Antoniono RJ, Ross HJ, Paisley TE, Oh Y (2006). Neuroendocrine-like differentiation of non-small cell lung carcinoma cells: regulation by cAMP and the interaction of mac25/IGFBP-rP1 and 25.1. Oncogene.

[33] Sato J, Hasegawa S, Akaogi K, Yasumitsu H, Yamada S, Sugahara K (1999). Identification of cell-binding site of angiomodulin (AGM/TAF/Mac25) that interacts with heparan sulfates on cell surface. J Cell Biochem.

[34] Evdokimova V, Tognon CE, Benatar T, Yang W, Krutikov K, Pollak M (2012). IGFBP7 binds to the IGF-1 receptor and blocks its activation by insulin-like growth factors. Sci Signal.

[35] Sprenger CC, Damon SE, Hwa V, Rosenfeld RG, Plymate SR (1999). Insulin-like growth factor binding protein-related protein 1 (IGFBP-rP1) is a potential tumor suppressor protein for prostate cancer. Cancer Res.

[36] Ruan W, Xu E, Xu F, Ma Y, Deng H, Huang Q (2007). IGFBP7 plays a potential tumor suppressor role in colorectal carcinogenesis. Cancer Biol Ther.

[37] Yamashita S, Tsujino Y, Moriguchi K, Tatematsu M, Ushijima T (2006). Chemical genomic screening for methylation-silenced genes in gastric cancer cell lines using 5-aza-2′-deoxycytidine treatment and oligonucleotide microarray. Cancer Sci.

[38] An W, Ben QW, Chen HT, Zheng JM, Huang L, Li GX (2012). Low expression of IGFBP7 is associated with poor outcome of pancreatic ductal adenocarcinoma. Ann Surg Oncol.

[39] Suzuki H, Igarashi S, Nojima M, Maruyama R, Yamamoto E, Kai M (2010). IGFBP7 is a p53-responsive gene specifically silenced in colorectal cancer with CpG island methylator phenotype. Carcinogenesis.

[40] Burger AM, Zhang X, Li H, Ostrowski JL, Beatty B, Venanzoni M (1998). Down-regulation of T1A12/mac25, a novel insulin-like growth factor binding protein related gene, is associated with disease progression in breast carcinomas. Oncogene.

[41] Sullivan L, Murphy TM, Barrett C, Loftus B, Thornhill J, Lawler M (2012). IGFBP7 promoter methylation and gene expression analysis in prostate cancer. J Urol.

[42] Komatsu S, Okazaki Y, Tateno M, Kawai J, Konno H, Kusakabe M (2000). Methylation and downregulated expression of mac25/insulin-like growth factor binding protein-7 is associated with liver tumorigenesis in SV40T/t antigen transgenic mice, screened by restriction landmark genomic scanning for methylation (RLGS-M). Biochem Biophys Res Commun.

[43] Mutaguchi K, Yasumoto H, Mita K, Matsubara A, Shiina H, Igawa M (2003). Restoration of insulin-like growth factor binding protein-related protein 1 has a tumor-suppressive activity through induction of apoptosis in human prostate cancer. Cancer Res.

[44] Shao L, Huang Q, Luo M, Lai M (2004). Detection of the differentially expressed gene IGF-binding protein-related protein-1 and analysis of its relationship to fasting glucose in Chinese colorectal cancer patients. Endocr Relat Cancer.

[45] Tomimaru Y, Eguchi H, Wada H, Kobayashi S, Marubashi S, Tanemura M (2011). IGFBP7 downregulation is associated with tumor progression and clinical outcome in hepatocellular carcinoma. Int J Cancer.

[46] Ouellet V, Zietarska M, Portelance L, Lafontaine J, Madore J, Puiffe ML (2008). Characterization of three new serous epithelial ovarian cancer cell lines. BMC Cancer.

[47] Wojnarowicz PM, Breznan A, Arcand SL, Filali-Mouhim A, Provencher DM, Mes-Masson AM (2008). Construction of a chromosome 17 transcriptome in serous ovarian cancer identifies differentially expressed genes. Int J Gynecol Cancer.

[48] Quinn MC, Wilson DJ, Young F, Dempsey AA, Arcand SL, Birch AH (2009). The chemiluminescence based Ziplex automated workstation focus array reproduces ovarian cancer Affymetrix GeneChip expression profiles. J Transl Med.

[49] Birch AH, Quinn MC, Filali-Mouhim A, Provencher DM, Mes-Masson AM, Tonin PN (2008). Transcriptome analysis of serous ovarian cancers identifies differentially expressed chromosome 3 genes. Mol Carcinog.

[50] Presneau N, Dewar K, Forgetta V, Provencher D, Mes-Masson AM, Tonin PN (2005). Loss of heterozygosity and transcriptome analyses of a 1.2 Mb candidate ovarian cancer tumor suppressor locus region at 17q25.1-q25.2. Mol Carcinog.

[51] Rozen S, Skaletsky H (2000). Primer3 on the WWW for general users and for biologist programmers. Methods Mol Biol.

[52] Karolchik D, Baertsch R, Diekhans M, Furey TS, Hinrichs A, Lu YT (2003). The UCSC genome browser database. Nucleic Acids Res.

[53] Zietarska M, Maugard CM, Filali-Mouhim A, Alam-Fahmy M, Tonin PN, Provencher DM (2007). Molecular description of a 3D in vitro model for the study of epithelial ovarian cancer (EOC). Mol Carcinog.

[54] Sjoblom T, Jones S, Wood LD, Parsons DW, Lin J, Barber TD (2006). The consensus coding sequences of human breast and colorectal cancers. Science.

[55] Thompson JD, Higgins DG, Gibson TJ (1994). CLUSTAL W: improving the sensitivity of progressive multiple sequence alignment through sequence weighting, position-specific gap penalties and weight matrix choice. Nucleic Acids Res.

[56] Birch AH, Arcand SL, Oros KK, Rahimi K, Watters AK, Provencher D (2011). Chromosome 3 anomalies investigated by genome wide SNP analysis of benign, low malignant potential and low grade ovarian serous tumours. PLoS One.

[57] Salvador S, Gilks B, Kobel M, Huntsman D, Rosen B, Miller D (2009). The fallopian tube: primary site of most pelvic high-grade serous carcinomas. Int J Gynecol Cancer.

[58] Auersperg N, Wong AS, Choi KC, Kang SK, Leung PC (2001). Ovarian surface epithelium: biology, endocrinology, and pathology. Endocr Rev.

[59] Zorn KK, Jazaeri AA, Awtrey CS, Gardner GJ, Mok SC, Boyd J (2003). Choice of normal ovarian control influences determination of differentially expressed genes in ovarian cancer expression profiling studies. Clin Cancer Res.

[60] Dubeau L (1999). The cell of origin of ovarian epithelial tumors and the ovarian surface epithelium dogma: does the emperor have no clothes?. Gynecol Oncol.

[61] Wilson EM, Oh Y, Rosenfeld RG (1997). Generation and characterization of an IGFBP-7 antibody: identification of 31kD IGFBP-7 in human biological fluids and Hs578T human breast cancer conditioned media. J Clin Endocrinol Metab.

[62] Wajapeyee N, Kapoor V, Mahalingam M, Green MR (2009). Efficacy of IGFBP7 for treatment of metastatic melanoma and other cancers in mouse models and human cell lines. Mol Cancer Ther.

[63] Jiang W, Xiang C, Cazacu S, Brodie C, Mikkelsen T (2008). Insulin-like growth factor binding protein 7 mediates glioma cell growth and migration. Neoplasia.

[64] Amemiya Y, Yang W, Benatar T, Nofech-Mozes S, Yee A, Kahn H (2011). Insulin like growth factor binding protein-7 reduces growth of human breast cancer cells and xenografted tumors. Breast Cancer Res Treat.

[65] Lee Y, Miron A, Drapkin R, Nucci MR, Medeiros F, Saleemuddin A (2007). A candidate precursor to serous carcinoma that originates in the distal fallopian tube. J Pathol.

[66] Crum CP, Drapkin R, Miron A, Ince TA, Muto M, Kindelberger DW (2007). The distal fallopian tube: a new model for pelvic serous carcinogenesis. Curr Opin Obstet Gynecol.

[67] Smith E, Ruszkiewicz AR, Jamieson GG, Drew PA (2014). IGFBP7 is associated with poor prognosis in oesophageal adenocarcinoma and is regulated by promoter DNA methylation. Br J Cancer.

[68] Adachi YYH, Itoh F, Arimura Y, Nishi M, Endo T, Imai K (2001). Clinicopathologic and prognostic significance of matrilysin expression at the invasive front in human colorectal cancers. Int J Cancer.

[69] Kashyap MK, Pawar HA, Keerthikumar S, Sharma J, Goel R, Mahmood R (2012). Evaluation of protein expression pattern of stanniocalcin 2, insulin-like growth factor-binding protein 7, inhibin beta A and four and a half LIM domains 1 in esophageal squamous cell carcinoma. Cancer Biomark.

[70] Umeda F, Ono Y, Sekiguchi N, Hashimoto T, Masakado M, Nakamura K (1998). Increased mRNA expression of a novel prostacyclin-stimulating factor in human colon cancer. J Gastroenterol.

[71] Domcke S, Sinha R, Levine DA, Sander C, Schultz N (2013). Evaluating cell lines as tumour models by comparison of genomic profiles. Nat Commun.

[72] Benatar T, Yang W, Amemiya Y, Evdokimova V, Kahn H, Holloway C (2012). IGFBP7 reduces breast tumor growth by induction of senescence and apoptosis pathways. Breast Cancer Res Treat.

[73] Singer G, Stohr R, Cope L, Dehari R, Hartmann A, Cao DF (2005). Patterns of p53 mutations separate ovarian serous borderline tumors and low- and high-grade carcinomas and provide support for a new model of ovarian carcinogenesis: a mutational analysis with immunohistochemical correlation. Am J Surg Pathol.

[74] Hwa V, Oh Y, Rosenfeld RG (1999). Insulin-like growth factor binding proteins: a proposed superfamily. Acta Paediatr Suppl.

[75] Lopez-Bermejo A, Buckway CK, Devi GR, Hwa V, Plymate SR, Oh Y (2000). Characterization of insulin-like growth factor-binding protein-related proteins (IGFBP-rPs) 1, 2, and 3 in human prostate epithelial cells: potential roles for IGFBP-rP1 and 2 in senescence of the prostatic epithelium. Endocrinology.

[76] Swisshelm K, Ryan K, Tsuchiya K, Sager R (1995). Enhanced expression of an insulin growth factor-like binding protein (mac25) in senescent human mammary epithelial cells and induced expression with retinoic acid. Proc Natl Acad Sci U S A.

[77] Hanrahan AJ, Schultz N, Westfal ML, Sakr RA, Giri DD, Scarperi S (2011). Genomic complexity and AKT dependence in serous ovarian cancer. Cancer Discov.

[78] Yuan ZQ, Sun M, Feldman RI, Wang G, Ma X, Jiang C (2000). Frequent activation of AKT2 and induction of apoptosis by inhibition of phosphoinositide-3-OH kinase/Akt pathway in human ovarian cancer. Oncogene.

[79] Cristiano BE, Chan JC, Hannan KM, Lundie NA, Marmy-Conus NJ, Campbell IG (2006). A specific role for AKT3 in the genesis of ovarian cancer through modulation of G(2)-M phase transition. Cancer Res.

[80] Okamura JHY, Moon D, Brait M, Chang X, Kim MS (2012). Downregulation of insulin-like growth factor-binding protein 7 in cisplatin-resistant non-small cell lung cancer. Cancer Biol Ther.

[81] Garnett MJ, Edelman EJ, Heidorn SJ, Greenman CD, Dastur A, Lau KW (2012). Systematic identification of genomic markers of drug sensitivity in cancer cells. Nature.

[82] Verhagen HJ, de Leeuw DC, Roemer MG, Denkers F, Pouwels W, Rutten A (2014). IGFBP7 induces apoptosis of acute myeloid leukemia cells and synergizes with chemotherapy in suppression of leukemia cell survival. Cell Death Dis.

[83] Severino V, Alessio N, Farina A, Sandomenico A, Cipollaro M, Peluso G (2013). Insulin-like growth factor binding proteins 4 and 7 released by senescent cells promote premature senescence in mesenchymal stem cells. Cell Death Dis.

