# Supplementary figures and images for: Low levels of IGFBP7 expression in high-grade serous ovarian carcinoma is associated with patient outcome
Source: BMC Cancer. 2015 Mar 17;15:135. doi: 10.1186/s12885-015-1138-8 (PMC4381406; doi:10.1186/s12885-015-1138-8)

A

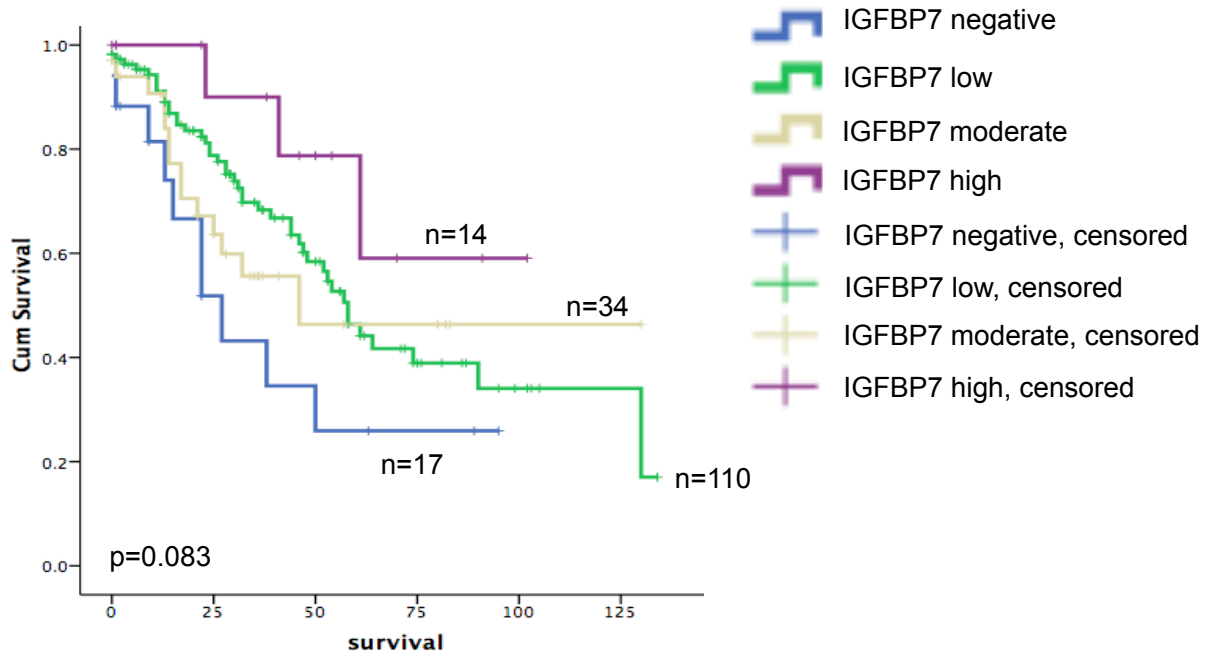

B

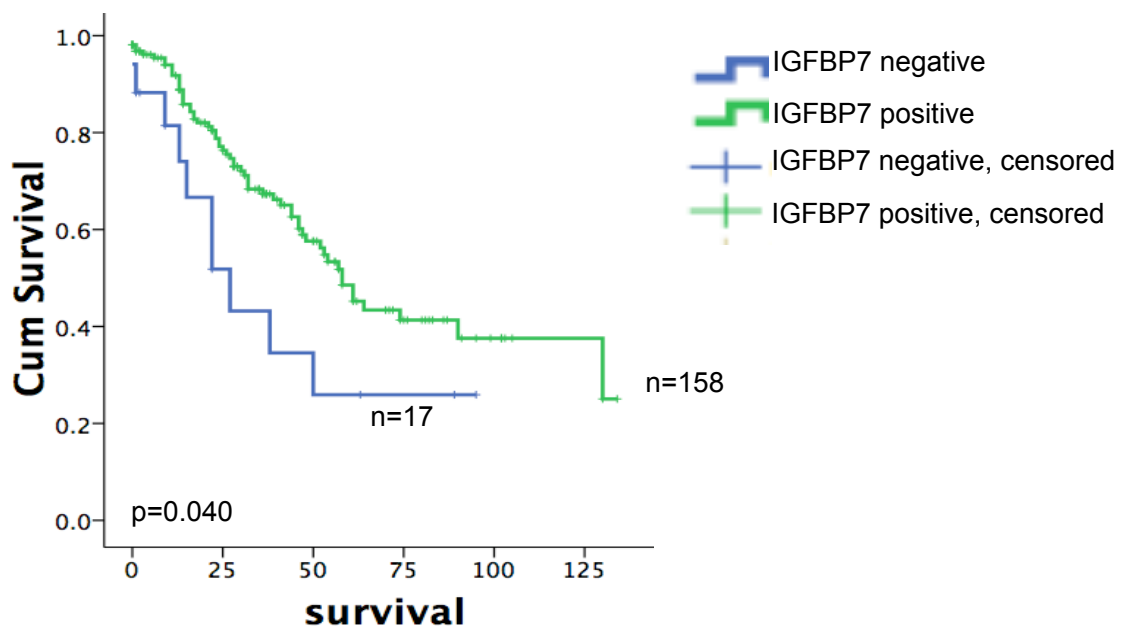

Supplement: Additional file 5: — Kaplan-Meier survival curve analyses of IGFBP7 staining intensity in HGSCs. A, Kaplan–Meier survival curve analysis of HGSC cases for overall survival of patients whose tumours showed negative (n = 17), low (n = 110), moderate (n = 34) and high (n = 14) IGFBP7 staining. B, Kaplan–Meier survival curve analysis of HGSC cases for overall survival of patients whose tumours showed negative (n = 17), and positive (n = 158) IGFBP7 staining. All p-values were derived from log-rank tests. [file 12885_2015_1138_MOESM5_ESM.pdf]

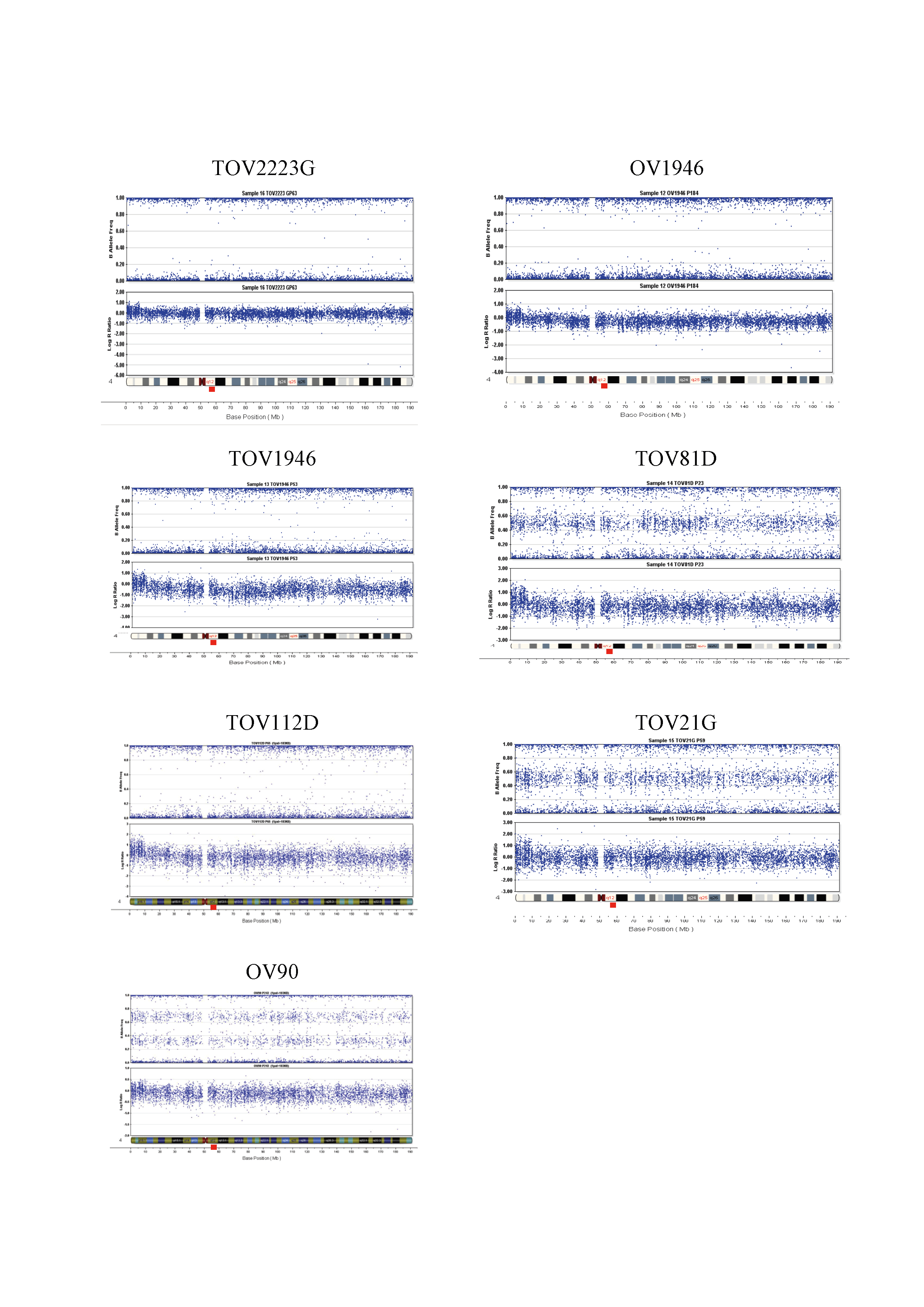

Supplement: Additional file 6: — SNP array imaging for chromosome 4 of the EOC cell lines. The top plot of each figure shows B allele frequencies for each chromosome 4 SNP marker aligned to its chromosomal position. The bottom plot of each figure contains the log R ratio, which provides an indication of the copy number for each SNP marker aligned to its chromosomal position and a red bar indicates IGFBP7 locus. Note the complete LOH of the entire chromosome 4 for TOV2223G, OV1946, TOV1946 and TOV112D. OV90 exhibits an allelic imbalance for the whole chromosome. [file 12885_2015_1138_MOESM6_ESM.tiff]

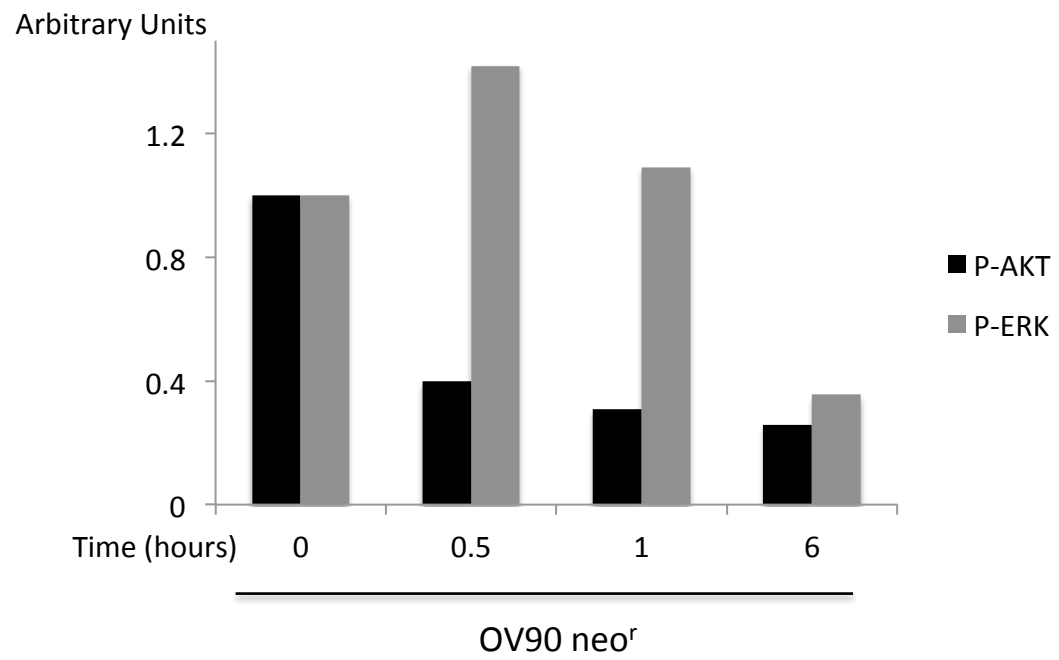

Supplement: Additional file 7: — Quantification of western blots results of P-AKT and P-ERK in OV90neorcells exposed to rIGFBP7. Band intensities were quantified using ImageJ software, normalized to untreated samples and expressed as arbitrary units relative to controls. [file 12885_2015_1138_MOESM7_ESM.pdf]
